# Activation of the motivation-related ventral striatum during delusional experience

**DOI:** 10.1038/s41398-018-0347-8

**Published:** 2018-12-18

**Authors:** Tuukka T. Raij, Tapani J. J. Riekki, Eva Rikandi, Teemu Mäntylä, Tuula Kieseppä, Jaana Suvisaari

**Affiliations:** 1 Department of Psychiatry, Helsinki University and Helsinki University Hospital, Helsinki, Finland; 2 Department of Neuroscience and Biomedical Engineering and Advanced Magnetic Imaging Center, Aalto University School of Science, Espoo, Finland; 3 Faculty of Medicine, Department of Psychology and Logopedics, University of Helsinki, Helsinki, Finland; 4 Mental Health Unit, National Institute for Health and Welfare, Helsinki, Finland; 5 Institute for Molecular Medicine Finland (FIMM), University of Helsinki, Helsinki, Finland

## Abstract

Delusion is the most characteristic symptom of psychosis, occurring in almost all first-episode psychosis patients. The motivational salience hypothesis suggests delusion to originate from the experience of abnormal motivational salience. Whether the motivation-related brain circuitries are activated during the actual delusional experience remains, however, unknown. We used a forced-choice answering tree at random intervals during functional magnetic resonance imaging to capture delusional and non-delusional spontaneous experiences in patients with first-episode psychosis (*n* = 31) or clinical high-risk state (*n* = 7). The motivation-related brain regions were identified by an automated meta-analysis of 149 studies. Thirteen first-episode patients reported both delusional and non-delusional spontaneous experiences. In these patients, delusional experiences were related to stronger activation of the ventral striatum in both hemispheres. This activation overlapped with the most strongly motivation-related brain regions. These findings provide an empirical link between the actual delusional experience and the motivational salience hypothesis. Further use and development of the present methods in localizing the neurobiological basis of the most characteristic symptoms may be useful in the search for etiopathogenic pathways that result in psychotic disorders.

## Introduction

Delusion is the most characteristic symptom of psychosis, occurring in about 96%^1^ of first-episode psychosis patients. Mapping the brain regions related to such a key symptom would be valuable for understanding the neurobiological basis of psychotic disorders. The brain correlates of delusion could be linked to upstream factors to elucidate etiopathogenic pathways as a target for better diagnostic and treatment methods^2^. Furthermore, unraveling the neuronal origins of delusion could help to assess the more general question about how the sense of reality is constructed in the human mind and brain.

Delusion is described in the DSM-5 as a fixed belief that is not amenable to change in light of conflicting evidence. Even though such a symptom is typically associated with thinking, early delusional experiences have been considered to be “something basic and primary which comes before thought”^3^. We therefore refer to sensations and feelings with delusional contents as well as to the delusional thoughts as “delusional experience”.

Delusions are thought to arise from interplay of multiple factors, including cognitive biases such as jumping to conclusion, distortions in predictive coding and processing of certainty, together with dysfunctional striatal dopaminergic signaling^4–6^. There is, however, strong support for the hypothesis that suggests dopaminergic dysfunction-related experience of abnormal motivational salience to be a final common pathway in formation of delusion^6–8^. Among brain structures, motivational salience has been most strongly associated with the ventral striatum^9^, but the cortical salience network has been linked to salience processing as well^10,11^. Whether this corticostriatal circuitry is activated during the actual delusional experience remains unknown.

Previous studies on the brain correlates of delusion have measured brain functioning during resting state and various stimuli without considering the actual experience of delusion during scanning. Severity of delusions as measured outside the scanner has been associated with decreased resting-state activity of the medial temporal lobe, the lateral prefrontal cortex, and the anterior cingulate cortex^12^ and with increased activity of the dorsal associative striatum^13^. Task studies on brain correlates of delusion have found delusion severity to correlate with activation in the brain regions that are typically activated during the particular task^14–25^. A study by Menon and coworkers is of particular interest here, as it aimed to provoke experiences similar to delusions of reference in patients with these symptoms. The authors presented statements about a person and asked how strongly a participant felt that the statement was specifically about him/her. In comparison with healthy control subjects, activation of the insula, cortical midline structures, and the ventral striatum was less dependent on the endorsement to the statements in the patients^18^. The relationship of these findings to the actual delusional experience is difficult to define, however, as the actual delusional experiences during scanning were not assessed^26^.

Measuring brain activation during the actual spontaneous delusional experience is enabled by experience sampling, i.e., asking a subject at random time points about the present experience. This method has been established in studies of normal experiences as well as delusions^27^ outside the scanner and has been implemented in brain imaging studies on spontaneous experiences in healthy subjects^28^. While the method has been used, to our knowledge, to investigate brain correlates of a symptom only in one study on verbal hallucinations^29^, related “symptom-capture studies”, based on self-reports of the beginning and end of the voices, have provided valuable insights into the brain correlates of chronic verbal hallucinations^30,31^. Similarly to the experience of hallucination, the delusional experiences are not constantly present, but alternate with delusion-free periods^27^, thus allowing within-subject comparisons of delusion *vs.* non-delusion periods.

Patients could be taught to recognize and report their delusional experiences, but this might interfere with the actual experience. Alternatively, delusions can be probed indirectly, by asking about contents of the experience that are likely indicative of delusion^27^. Such indicators are suggested by the high prevalence of the persecutory delusion and the delusion of reference, being present in 74 and 67% of first-episode patients, respectively^1^. Persecutory delusions are experiences of others intending to harm oneself, and delusions of reference are defined as experiences of being “observed, followed, discussed, or the topic of messages or communications”^32^ by others. Thus, a common denominator of most delusional contents in first-episode psychosis is intentions of others focused on oneself. By assessing this and other typical delusional contents in an experience sampling paradigm during functional magnetic resonance imaging (fMRI), we aimed to map the brain functioning during actual spontaneous delusional experience with a focus on corticostriatal salience circuitries. If delusions arise as a cognitive effort to make sense of the experience of aberrant motivational salience, as proposed by the hypothesis of motivational salience^8^, early delusional experiences should be accompanied by the activation of the brain substrates of motivation.

## Materials and methods

### Participants

Thirty-one patients with first-episode psychosis (FEP) and seven clinical high-risk patients, aged 18–40 years, were recruited from the catchment area of the Helsinki University Hospital and the City of Helsinki, Finland, between November 2010 and September 2015. Clinical high-risk patients were pooled with the FEP patients to assess early delusional experiences. All primary psychotic disorders were included, whereas substance-induced psychoses and psychotic disorders due to a general medical condition were excluded. The inclusion criterion for the FEP patients was a score of at least 4 on items assessing delusions (Unusual Thought Content) or hallucinations in the Brief Psychiatric Rating Scale—Extended (BPRS-E)^33^. Healthy control subjects were included to validate the answer options that were planned to be indicative of delusion: if certain answers were selective to delusional experiences they should be more frequent in the patients than in healthy control subjects. Twenty-six healthy control subjects, matched by age and sex, were identified from the Population Register Center and recruited as controls to a larger Helsinki early psychosis study. In addition, 32 control subjects were recruited to the experience sampling study by word of mouth among the investigators’ contacts. The participants of the Helsinki early psychosis study were evaluated thoroughly at baseline and at follow-up. The additional 32 control subjects were asked about severe mental or somatic health problems at baseline for exclusion purposes.

The study protocol was approved by the Ethics Committee of the Hospital District of Helsinki and Uusimaa and by the Institutional Review Boards of the Hospital District of Helsinki and Uusimaa, the City of Helsinki, and the National Institute for Health and Welfare. All participants gave written informed consent after having received a complete description of the study.

### Clinical assessment

Diagnostic assessment, based on the research version of the Structured Clinical Interview for DSM-IV^34^, was conducted on the participants of the Helsinki early psychosis study. One of the authors (JS) also reviewed all psychiatric medical records of the patients for the final diagnostic assessment at the two-months follow-up. One high-risk patient was excluded from the analysis due to non-psychotic diagnosis (obsessive compulsive disorder) at the follow-up.

We used the BPRS-E^33^ complemented by the global ratings of Alogia, Anhedonia-Asociality, and Avolition-Apathy from the Scale for the Assessment of Negative Symptoms (SANS)^35^ to assess the severity of symptoms. Unusual thought content was used to assess delusion severity. The sum score of BPRS items Hallucinations, Unusual thought content, Bizarre behavior, and conceptual disorganization was used for the overall severity of positive symptoms. The severity of negative symptoms was assessed with a sum score of BPRS item Blunted affect and SANS ratings of Alogia, Anhedonia-Asociality, and Avolition-Apathy. BPRS items were rescaled from 1–7 to 0–6 (similar to SANS) for calculating the sum scores. In addition, the total score for BPRS-E was calculated as a sum score of all items. For the assessment of current functioning, we used the Global Assessment of Functioning (GAF). Information about medication was based on the combination of the patient report at the baseline interview and the medical records.

### Experience sampling

**Fig. 1:**
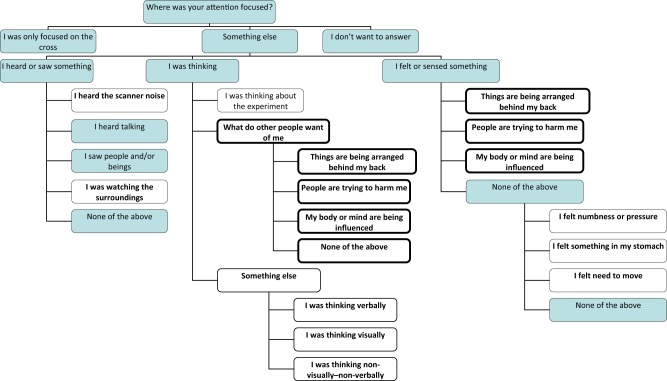
Answer tree. Selected options were followed by the next options as shown by the lines. In the main analysis, the likely delusional options in bold boxes with white background were contrasted with the other options with white background. Answers not included in the comparison are shown on a dark background

The task was introduced and practiced on a computer before entering the scanner. We asked the participant to attend to a fixation cross. We explained that other percepts or thoughts may still capture one’s attention, and asked them to select, from the response options the category best matching the primary focus of their attention during previous seconds. These options (Fig. 1) were presented at random intervals between 12 and 40.5 s with Presentation software and projected to a screen inside the scanner. The response option to the right was highlighted by pressing the right-hand button and the one to the left by pressing the left-hand button. The answer was recorded two s after the last button press. Answering options were based on literature on spontaneous experiences^28^ and typical symptoms of psychotic disorders^1,34^. Participants selected one among three to five answer alternatives to each of one to four questions during each answering period (Fig. 1).

Participants were asked to repeat the fMRI session if there was scanning time remaining. Due to time limitations only four patients completed the second session, whereas 34 patients completed one session. In addition, we included behavioral data from one session of 58 healthy control subjects of similar age and sex to estimate the relevance of the delusion probes.

### Imaging

We acquired 600 blood-oxygenation-level-dependent (BOLD) functional images for each functional echo planar imaging session using a Siemens Magnetom Skyra 3-T system (Siemens AG, Erlangen, Germany) with a 30-channel head coil at the Aalto University Advanced Magnetic Imaging Center. Imaging parameters for the functional images were as follows: TR, 1500 ms; TE, 30 ms; flip angle, 75°; field of view, 24 cm; base resolution 64 × 64; and 36 slices resulting in a voxel size of 3.75 × 3.75 × 4 mm. In addition, T1-weighted structural images were acquired with a voxel size of 1 × 1 × 1 mm.

### Preprocessing

Functional images were corrected for slice timing and realigned by linear translation and rotation for movement correction. We co-registered the functional images with the anatomical ones and used non-linear transformation matrices, based on the anatomical images, to normalize functional images to the Montreal Neurological Institute Template. Normalized images were smoothed with a Gaussian kernel of 8 mm full-width at half-maximum.

### Analysis

We tested for group differences between age, sex, and likely delusional experiences during the first imaging session in IBM SPSS. We included in the main fMRI analysis 13 patients who reported both delusional and non-delusional experiences during imaging. We modeled the experience-related brain activation during two time points preceding the experience probe and during the first time point of the answering period within SPM12 general linear model (http://www.fil.ion.ucl.ac.uk/spm/software/spm12/). The model was not convolved with hemodynamic response function to avoid overlap of experience and answering periods as otherwise some of a strong answering-related activation might be erroneously modeled in an overlapping experience regressor. Assuming a hemodynamic delay of 5 s, the model best captures brain activation 3.5–8 s before the answering period. Previous experience sampling studies in healthy subjects suggest the experience in this time window to be consistent with the consequent answer about the experience^36^. We computed contrast images between the experiences labeled as delusional *vs.* experiences labeled as non*-*delusional. The contrast images were entered to a permutation test in SnPM-extension of SPM with 5000 permutations (http://warwick.ac.uk/snpm)^37^. We repeated the same analysis with antipsychotic equivalent doses as a nuisance covariate due to a possible effect of antipsychotic medication on the salience circuitries. In addition, we repeated the analysis with contrast images computed with movement parameters as a nuisance covariate as head movement could contribute to findings. Antipsychotic equivalent doses were computed according to Leucht and coworkers^38^ and three translation and three rotation movement parameters were derived from SPM realignment.

We conducted an automated meta-analysis of 149 motivation-related brain imaging studies in Neurosynth (http://www.neurosynth.org/)^39^ to further test the suggested link of the neuronal correlates of delusion to the processing of motivation^8^. The meta-analysis assessed the brain regions more strongly associated with the term “motivational” than with the other terms.

### Statistical methods

Activation was familywise error-corrected for multiple comparisons in the whole brain and in the regions of interest (ROI) at the voxel level and at the cluster level. We used the voxelwise cluster-defining threshold *p* < 0.01, which has been shown to reasonably control for false positives in a permutation test^40^. We modeled the ventral striatal ROI as a sphere with an 8-mm radius, centered at the mean coordinates of the two nearby maxima that were related to abnormal functioning in patients with delusion of reference in the study of Menon and co-workers (*x*, *y*, *z* = −20, 13, −4; Supplementary Fig. 1)^18^. As the striatal dysfunction in psychosis is likely bilateral, we tested also activation in the right hemisphere homolog area (sphere with 8-mm radius, centered at the *x*, *y*, *z* = 20, 13, −4; Supplementary Fig. 1). Core regions of the cortical salience network, comprising the left and the right anterior insula (two ROIs) and the bilateral dorsal anterior cingulate cortex-supplementary motor cortex (one ROI), were derived from the Functional Imaging in Neuropsychiatric Disorders Lab website (findlab.stanford.edu were/functional_ROIs.html)^41^. The automated meta-analysis of motivation was thresholded at the Neurosynth default (*p* < 0.01, false discovery rate corrected for multiple comparisons).

We also extracted activation strengths in delusion *vs*. non-delusion contrast in each striatal ROI and correlated them with delusion severity. One-tailed Spearman’s correlation was used due to *a priori* assumption of positive correlation, i.e., stronger activation strengths to be associated with more severe delusion.

### Control analysis

As the time points during delusional experience were limited, we tested whether any brain activation can be detected with the present methods and the present amount of data. This control analysis was conducted with the same 13 patients who were included in the main analysis and contrasted visual answering probes *vs.* rest periods. The answering periods were modeled as time points from five to seven (i.e., 6−10.5 s) after the presentation of the experience probe to adjust for the hemodynamic delay. The number of answering-related time points was matched individually to the number of delusion-related time points, and the number of rest-related time points was matched individually to the number of the non-delusion-related time points. Corresponding to the main analysis, the model regressors were modeled without a hemodynamic lag, and the same statistical thresholds were used. As the experience probes were presented visually, we modeled ROIs as 8-mm radius spheres in the left and right primary visual cortex, centered at the coordinates of the statistically most significant regional activation in an automated meta-analysis^39^ of the association of brain activation with the word “visual” (*x*, *y*, *z* = 12, −86, 0 and −6, −88, 2; Supplementary Fig. 1).

## Results

### Behavioral findings

The healthy control subjects (mean (S.D.) 27.6 (5.1) years, 72% males) did not differ from the patients (26.1 (5.4) years, 71% males) in age (*p* = 0.17, independent-samples *t* test) or sex (*p* = 0.89, Chi-square test). At the baseline assessment, typically one to two weeks after inclusion, the patients had delusion score 3.7 (1.8) and hallucination score 2.3 (1.7), delusion score being higher or equal to the hallucination score in all patients.

Patients and healthy control subjects completed mean (S.D.) 21.9 (4.4) and 22.9 (2.8) (*p* = 0.17, independent samples *t*-test) answering periods of 16.0 s (6.9 s) and 13.9 s (4.4 s) (*p* = 0.10) during a 15-min fMRI session. During the first imaging session 14 of the 37 patients and one of the 58 healthy control subjects reported likely delusional experiences (*p* < 0.001, Chi-square test). Thirteen of these patients reported non-delusional thoughts, sensations or feelings, allowing intra-individual comparison of delusional *vs*. non-delusional experience. Characteristics of the 13 patients are presented in Table 1. The most prominent symptom in these patients in the BPRS-E interview was delusion, with a mean score of 3.8 (range 1–7) out of 7.

**Table 1:** Demographic and clinical characteristics of the patients included in the final analysis

Characteristic	*n*	%
Female	4	31
Schizophrenia	2	15
Schizophreniform disorder	3	23
Bipolar I disorder with psychotic features	1	8
Major depressive disorder with psychotic features	1	8
Brief psychotic disorder	1	8
Psychotic disorder not otherwise specified	5	38
	Mean	S.D.
Age	24.7	4.9
BPRS-E total score	43.9	10.2
Positive symptoms	6.0	4.0
Negative symptoms	5.6	4.0
GAF	39.4	8.9
Antipsychotic equivalent dose (mg)^a^	297	238

^a^Median 298 mg, range 0–684 mg. Main antipsychotic drug was olanzapine in five, risperidone in three, quetiapine in three, and aripiprazole in one patient. One patient didn’t use antipsychotic medication

These patients reported a mean of 2.8 (range 1–9, total 37) experiences with delusional contents during imaging. In ten subjects, all of the delusional experiences were reported as thoughts, and in three subjects the delusional experiences included sensations or feelings with delusional contents. Table 2 presents the contents of the delusional experiences. The comparison conditions included experiences of thinking (mean 8.2, range 1–18 per patient) and experiences of sensations or feelings (mean 1, range 0–6 per patient). The number of patients who reported feelings or sensations in addition to thoughts was similar among the experiences with delusional contents (3/13) and among the experiences without delusional contents (4/13).

**Table 2:** Contents of the experiences labeled as delusional

Probe	Subjects	Experiences^a^	
	*n*	Mean	Range
What do others want of me	10	2.2	1–5
Things being arranged behind my back	1	1	
Others intending to harm	2	4	
Mind or body being manipulated	3	3.3	1–7

^a^More than one category are reported by three patients

### Association of the delusional experience with brain activation

**Fig. 2:**
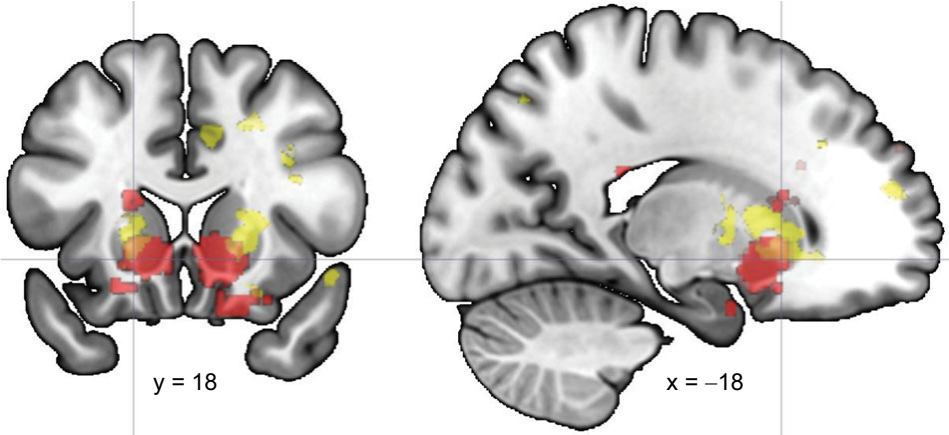
Brain activation related to the spontaneous delusional experience in comparison with the spontaneous experience in general overlaid with the motivation-related brain activation. Motivation-related activation from an automated meta-analysis (red) is thresholded at *p* < 0.01 (false discovery rate corrected), and the delusion-related activation (yellow) is thresholded at *p* < 0.01 (uncorrected) for visualization. Crosshair points the statistically most significant delusion-related activation

Experiences labeled as delusional were associated with stronger activation than other thoughts, sensations, and feelings in the bilateral ventral striatum (corrected for multiple comparisons within the volume of the left and the right ventral striatal ROI; Fig. 2; Table 3). This finding survived correction for antipsychotic dose equivalents (*p*-values for cluster-level and voxel-level inference 0.03 and 0.02 for the left and 0.004 and 0.06 for the right striatum) and for movement (*p*-values for cluster-level and voxel-level inference 0.006 and 0.008 for the left and 0.006 and 0.01 for the right striatum). The activation was statistically most significant in the co-ordinates labeled most frequently as ventral striatum (according to Neurosynth, *Z* = 8.2 for association between *x*, *y*, *z* = –18, 18, –6 and term “ventral striatum”). No activation survived correction for multiple comparisons in the whole-brain volume or in the volume of cortical salience network ROIs. Activation strengths in delusion *vs.* control condition correlated with delusion severity in the right striatum ROI (Spearman’s rho = 0.50, *p* = 0.04).

**Table 3:** Brain activation related to the spontaneous delusional experience in comparison with the spontaneous experience in general

Region	*p* voxel	Extent^a^	*p* cluster	*x*, *y*, z
Left ventral-rostral striatum	0.01	48	0.006	–18, 18, –6
Right ventral-rostral striatum	0.05	38	0.002	24, 9, –2

^a^Extent refers to the number of contiguous 3 × 3 × 3 mm voxels with *p* < 0.01 each within a region of interest

### Control analysis

Contrast of visually cued answering periods *vs.* rest periods with the same amount of data as in the main analysis showed highly significant activation in each region of interest (*p*-values for cluster-level and voxel-level inference 0.009 and 0.0006 for the left primary visual cortex and 0.002 and 0.004 for the right primary visual cortex, corrected for multiple comparisons; Supplementary Fig. 2).

## Discussion

We found delusional experiences to be associated with activation of the ventral striatum. This area overlapped with the most strongly motivation-related brain regions in an automated meta-analysis^39^. Our findings provide the first brain correlates of the actual experience of delusion and link such experience to the motivation-related brain activation^8^.

The relatively symmetrical bilateral distribution of the striatal activation suggests accuracy of the localization. The activation that was statistically significant at the voxel level is located in the coordinates that are commonly labeled as the ventral striatum in human neuroimaging studies. Functional, structural, and molecular imaging methods are available to focus on these regions to access structural, functional, and directional (effective) connectivity as well as molecular markers in patients with delusions. Such methods allow further investigation of the potential factors contributing to the delusion-related activation and may thus help to elucidate etiopathogenic pathways of the psychotic disorders.

Although we did not measure dopamine in this study, several lines of evidence suggest a link between the present findings and dopaminergic signaling. Dopamine is a prominent neurotransmitter in motivation-related functioning of the ventral striatum^9^, and striatal dopaminergic dysfunction in psychosis is well known. The dopamine-receptor-2-blocking drugs that alleviate psychosis have their main target in the striatum^42^, and striatal dopamine synthesis and release are elevated in patients with psychosis and subjects at risk of psychosis^43^. In our study, the antipsychotics had practically no effect on the findings. This might be because in the patients having acute delusional experiences the striatal dysfunction is still present despite medication, either due to insufficient blocking of the dopamine receptors or due to non-dopaminergic mechanisms (that may be however a persistent consequence of prior dopaminergic dysfunction). Present co-ordinates of the delusion-related striatal activation may help to focus the further search for the pathways between dopamine dysfunction and the delusional experience.

The localization of the delusion-related activation provides a new layer of evidence for the aberrant motivational salience hypothesis^8^. Motivational salience is strongly associated with the ventral striatum^9^, and the statistically most significant delusion-related voxels overlapped with the regions that were most strongly associated with motivation in an automated meta-analysis. The ventral striatum is activated during drug-cue presentation in addiction, with the activation strength correlating with self-reported craving^44^. This is of interest in the context of psychosis, as drug craving is known to override rational thinking. While in addiction the non-adaptive motivational salience is drug-bound, in early psychosis it may be arbitrary. Such a subjectively unexplained motivational salience may then be explained in a delusional manner by ways offered by one’s personal experiences and culture.

Considering multiple risk factors and brain alterations in psychotic disorders, it is unlikely that the striatal dopaminergic dysfunction is primary. It has been suggested that developmental alterations of the prefrontal cortex-striatal circuitries result in prefrontal hypo-functioning-related cognitive and negative symptoms and dysregulation of the striatal dopaminergic functioning that is manifested as positive symptoms^45,46^. In addition, animal models suggest hippocampal dysfunction to result in the striatal dopaminergic dysfunctioning^47^. Localizing the delusion-related striatal dysfunction may help further research on such up-stream pathways.

The relatively small data set and uncertainty about the subjective experience are the main limitations of this study. Due to limited amount of suitable subjects and experiences, “symptom capture” studies suffer typically from small power to detect activation. It is to be noted, however, that despite this limitation these studies have resulted in considerable advances in scientific understanding of chronic hallucinations^48^. While small sample size limits correction for multiple comparisons in the whole brain volume, our control analysis suggests power of the present study to be sufficient to detect brain activation in a region-of-interest analysis. It is to be noted, however, that effect size and variance may differ between the main and the control analysis and therefore the control analysis should be interpreted with caution. On the other hand, assuming independency between the striatal ROIs in the left and in the right hemispheres, it is unlikely to observe such a bilateral activation by chance. This together with the highly significant bilateral activation in the control analysis suggests that the observed activation may not be a false positive, and should be assessed in further studies. The answer options were carefully selected to capture the most typical contents of delusional experience, and these experiences were far more common in patients than in control subjects, supporting the delusional nature of the experience. Furthermore, activation strengths correlated with delusion severity in the right striatum. Further studies should, however, consider interviewing patients after scanning to exclude possible misunderstandings.

Our sample was diagnostically heterogeneous, but it is to be noted that different psychotic disorders can be treated with antidopaminergic medication. Among psychotic disorders it might thus be reasonable to assume that the brain basis of delusion shares similarities in the striatal functioning across current diagnostic categories.

## Conclusion

Our findings suggest that the actual delusional experience is associated with activation of the regions of the ventral striatum that are strongly linked to motivation. Interestingly, functioning of similar regions may override rational thinking in dependency disorders^44^. Multiple pathways may result in an unbalanced brain functioning, where the reasoning becomes biased by dysfunction of the machinery of motivation. The emerging understanding of the brain substrates of the most characteristic symptom of psychosis may help to elucidate such pathways.

## Supplementary information

Supplementary information
