# Supplementary material for: Activation of the motivation-related ventral striatum during delusional experience
Source: Transl Psychiatry. 2018 Dec 18;8:283. doi: 10.1038/s41398-018-0347-8 (PMC6298954; doi:10.1038/s41398-018-0347-8)
Supplement: Supplementary file 1 — Supplementary information [file 41398_2018_347_MOESM1_ESM.docx]

**Supplementary information**

Raij et al. Activation of the motivation-related ventral striatum during delusional experience

Supplementary Figures 1–2.

**
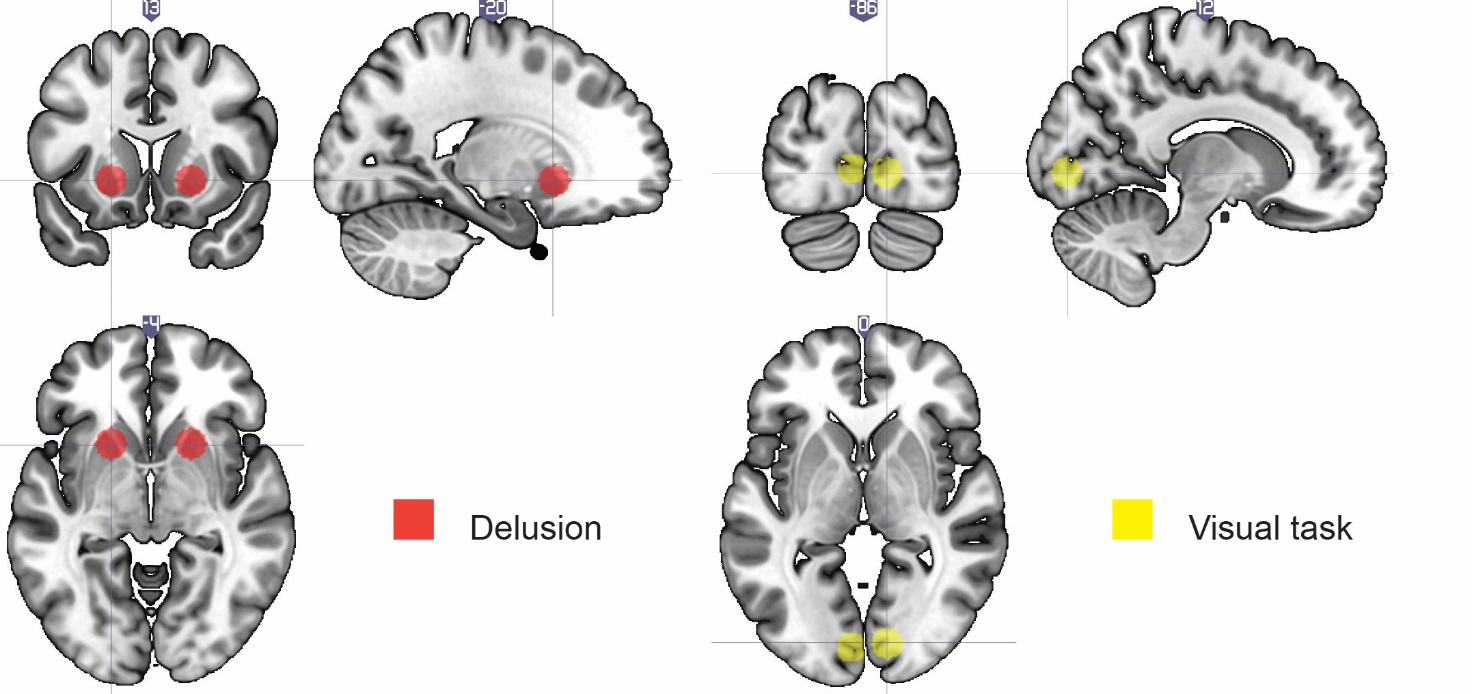
**

**Supplementary Fig. 1.** Regions of interest for the main analysis (left) and for the control analysis (right). Regions of interest for the main analysis included the cortical salience network (not shown) in addition to the ventral striatum (red).


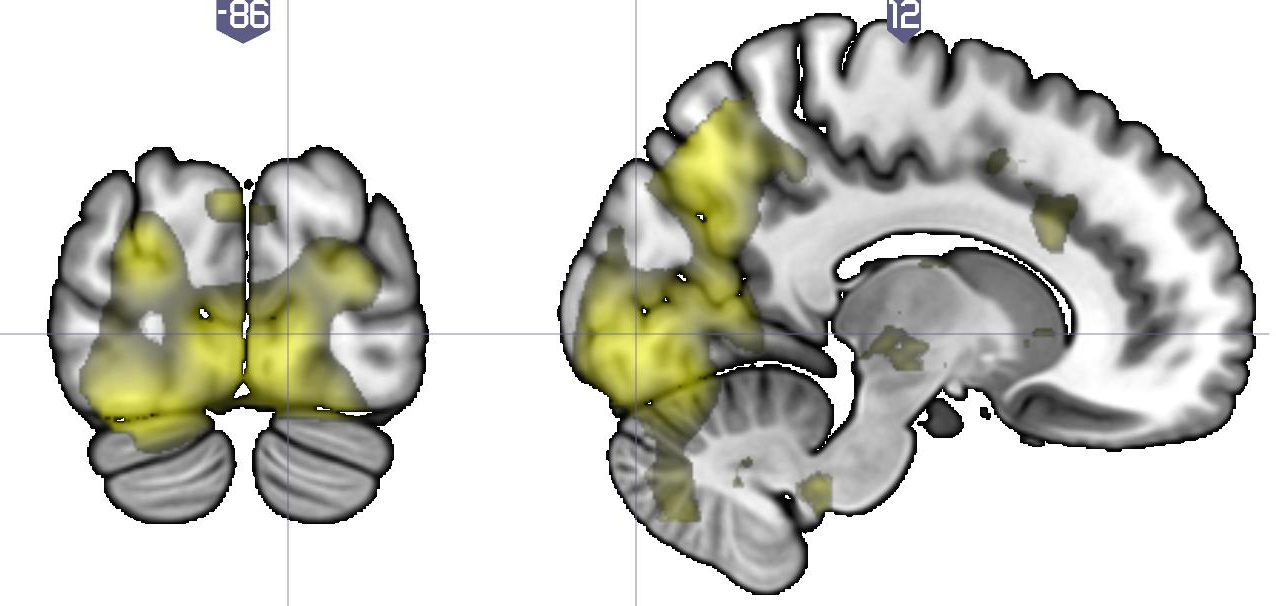


**Supplementary Fig. 2.**  Activation in the control analysis that compared visually cued answering periods with rest periods. Activation is shown in the whole brain without masks with the primary threshold corresponding to uncorrected *p* < 0.01. Crosshair points the center of the region of interest presented in the Supplementary Figure 1.
